# Renal Outcomes Following Autologous Stem Cell Transplantation in Dialysis‐Dependent Patients With Multiple Myeloma: A Single‐Center Study From a Low‐ and Middle‐Income Country

**DOI:** 10.1155/crh/1808249

**Published:** 2026-09-14

**Authors:** Maher Battat, Ahmad Khursani, Mohammedkhaled J. Alhendi, Momen Sawafta, Asa’d A. M. Daqa, Sa’ed H. Zyoud, Mohanad Hassan, Hisham Asaad, Riad Amer, Ahmed Enaya

**Affiliations:** ^1^ Bone Marrow Transplantation and Leukemia Unit, An-Najah National University Hospital (NNUH), Nablus 44839, State of Palestine; ^2^ Department of Internal Medicine, An-Najah National University Hospital, Nablus, State of Palestine, najah.edu; ^3^ Department of Medicine, Faculty of Medicine and Allied Medical Sciences, An-Najah National University, Nablus, State of Palestine, najah.edu; ^4^ Department of Clinical and Community Pharmacy, Faculty of Pharmacy, An-Najah National University, Nablus 44839, State of Palestine, najah.edu; ^5^ Clinical Research Center, An-Najah National University Hospital, Nablus 44839, State of Palestine, najah.edu; ^6^ Department of Nephrology, An-Najah National University Hospital, Nablus, State of Palestine, najah.edu; ^7^ Department of Hematology and Oncology, An-Najah National University Hospital, Nablus, State of Palestine, najah.edu

**Keywords:** autologous stem cell transplantation, case series, dialysis dependence, end-stage renal disease, hemodialysis, multiple myeloma, renal outcomes

## Abstract

**Background:**

Multiple myeloma (MM) frequently causes renal impairment, and a subset of patients progresses to end‐stage renal disease (ESRD) requiring dialysis. Dialysis dependence has historically been considered a relative contraindication to high‐dose conditioning and autologous stem cell transplantation (ASCT). Emerging evidence indicates that ASCT may provide effective hematologic control and, in selected patients, renal recovery.

**Methods:**

We conducted a retrospective case series of five dialysis‐dependent patients with MM who underwent ASCT at An‐Najah National University Hospital. Demographic and clinical characteristics, transplant variables, supportive‐care measures, infectious and noninfectious complications, hematologic recovery, admission and discharge laboratory values, and short‐term outcomes were extracted from the medical records.

**Results:**

The five patients had a mean age of 56 years and received reduced‐dose melphalan (140 mg/m^2^), followed by peripheral‐blood CD34+ cell infusion. The mean CD34+ collection and infusion doses were 4.3 × 10^6^/kg and 4.1 × 10^6^/kg, respectively. All patients achieved engraftment; mean neutrophil and platelet engraftment occurred on Days 13.0 and 19.4, respectively. Four patients (80%) developed neutropenic fever. *Clostridioides difficile* and *Staphylococcus epidermidis* were the most frequently documented pathogens. Two patients developed central line–associated bloodstream infection and septic shock. No 100‐day treatment‐related mortality occurred. Creatinine and blood urea nitrogen remained elevated, and all patients continued maintenance hemodialysis at discharge.

**Conclusions:**

ASCT was feasible in carefully selected dialysis‐dependent patients with MM and was associated with timely hematologic recovery and no 100‐day treatment‐related mortality. Infectious complications and vascular‐access management remain major clinical concerns. Larger prospective studies are needed to define long‐term renal outcomes and predictors of dialysis independence.

## 1. Background

Multiple myeloma (MM) is a clonal plasma‐cell malignancy and an important cause of cancer‐related morbidity and mortality [1, 2]. Renal impairment is among its most serious clinical complications. It may result from light‐chain cast nephropathy, hypercalcemia, infection, volume depletion, and treatment‐related nephrotoxicity. Renal dysfunction is associated with poorer outcomes and complicates treatment selection because several antimyeloma agents and conditioning regimens require dose adjustment or may increase toxicity [3, 4]. A subset of patients progresses to end‐stage renal disease (ESRD) requiring long‐term dialysis. Historically, dialysis dependence has been considered a marker of severe disease and a relative contraindication to high‐dose chemotherapy followed by autologous stem cell transplantation (ASCT) [5].

Although renal failure previously limited access to intensive therapy, evidence from retrospective cohorts supports the feasibility of ASCT in selected patients with MM and severe renal impairment [6]. Dialysis‐dependent patients can undergo ASCT with individualized melphalan dosing and careful supportive care, with acceptable toxicity and timely hematologic recovery [7, 8]. Some studies have also reported renal recovery or dialysis independence after ASCT in selected patients [7]. These findings suggest that, in selected patients, effective control of MM may provide hematologic benefit and may permit renal improvement.

Nevertheless, important evidence gaps remain. Most available studies are retrospective, include small and heterogeneous populations, and use inconsistent definitions and assessment times for renal recovery and dialysis independence [4]. In addition, clinically relevant peritransplant factors including conditioning‐regimen dose, dialysis timing, vascular‐access management, infection, transfusion support, and light‐chain kinetics are often incompletely reported [9–11]. This limits the development of standardized, evidence‐based recommendations for practice.

In this context, detailed institution‐level and patient‐level reports remain valuable. They provide practical insight into real‐world care, identify potentially modifiable risks such as central line‐associated bloodstream infection, and help describe the relationship between hematologic control and renal outcomes [12]. Such data complement larger cohorts, particularly in resource‐limited settings where treatment logistics and supportive‐care capacity may influence outcomes.

Given the evolving evidence, this study aimed to describe the clinical course of five dialysis‐dependent patients with MM who underwent ASCT at a tertiary hospital in a low‐ and middle‐income country. We report transplant feasibility, short‐term safety, hematologic recovery, infectious and noninfectious complications, and renal status during the index hospitalization [13–15]. Because dialysis‐dependent patients with MM are commonly underrepresented in clinical trials, detailed case series can provide practical information for transplant programs managing this high‐risk population.

## 2. Methods

### 2.1. Study Design and Setting

This retrospective case series was conducted at An‐Najah National University Hospital (NNUH), a tertiary hospital in Palestine. We reviewed consecutive adult patients with MM and dialysis‐dependent ESRD who underwent ASCT during 2021–2025. All eligible patients identified during the study period were included to reduce selection bias. The case series comprised five consecutive eligible patients. Reporting was guided by the Joanna Briggs Institute (JBI) Critical Appraisal Checklist for Case Series, which is provided as Supporting File 1.

### 2.2. Participants

Eligibility criteria were (1) MM diagnosed according to International Myeloma Working Group criteria; (2) maintenance hemodialysis dependence at the time of ASCT; and (3) completion of ASCT with sufficient medical‐record data for the prespecified variables. Five patients met these criteria and were included in the analysis.

### 2.3. Transplant Protocol and Peritransplant Care

All patients received reduced‐dose melphalan (140 mg/m^2^) because of dialysis‐dependent ESRD. Peripheral blood was the source of CD34+ cells. The local hydration protocol consisted of intravenous 0.9% saline at 75 mL/h for 2 h before and 2 h after melphalan administration. Antimicrobial prophylaxis followed institutional ASCT protocols and included levofloxacin or ciprofloxacin for antibacterial prophylaxis, acyclovir for antiviral prophylaxis, and fluconazole or itraconazole for antifungal prophylaxis during the neutropenic period. Febrile neutropenia was managed promptly with broad‐spectrum intravenous antibiotics, which were subsequently modified according to clinical and microbiological findings.

Filgrastim (granulocyte colony‐stimulating factor [G‐CSF]) was administered routinely from day + 1 at 5–10 μg/kg/day and continued until neutrophil engraftment. Irradiated packed red blood cells and platelet transfusions were administered according to clinical indications and institutional thresholds. Hemodialysis was coordinated with the nephrology team and scheduled approximately 24 h before stem‐cell infusion; stem‐cell reinfusion was performed 48 h after melphalan administration. Patients were monitored by a multidisciplinary team that included hematologists, nephrologists, infectious‐disease specialists, and transplant nurses throughout the transplantation period [16]. No backup stem‐cell product was used. Previous antimyeloma regimens included bortezomib–lenalidomide–dexamethasone (VRD), bortezomib–cyclophosphamide–dexamethasone (VCD), and bortezomib‐based therapy. No patient had received radiotherapy before ASCT.

### 2.4. Data Collection and Outcome Definitions

Data extracted from the hospital records included age, sex, comorbidities, previous antimyeloma therapy, duration of dialysis dependence, time from diagnosis to ASCT, vascular access, CD34+ cell collection and infusion doses, admission and discharge laboratory values, transfusion requirements, growth‐factor support, infectious and noninfectious complications, ICU admission, mechanical ventilation, and 100‐day treatment‐related mortality. Follow‐up duration was calculated from ASCT to the last documented clinical contact. Dialysis dependence was defined as the need for maintenance hemodialysis for at least 3 months before ASCT. All patients received maintenance hemodialysis through permanent tunneled central venous catheters. Because the cohort included only five patients, the analysis was descriptive; categorical variables are reported as *n* (%), and continuous variables as the mean (range).

Platelet engraftment was defined as the first of three consecutive days with a platelet count ≥ 20 × 109/L without platelet transfusion. Neutrophil engraftment was defined as the first of three consecutive days with an absolute neutrophil count ≥ 0.5 × 109/L.

### 2.5. Hematologic Disease‐Response Assessment

Pre‐ and post‐transplant disease response was recorded only when explicitly documented by the treating hematology team in the medical record. Serum free light‐chain testing was not consistently available, and the available records did not contain a sufficiently complete set of serial alternative IMWG measures to support independent retrospective assignment or reclassification of formal response categories. Accordingly, the depth of myeloma response was not analyzed as a study outcome, and the present analysis focused on engraftment, complications, short‐term mortality, and renal status.

### 2.6. Ethical Considerations

The study was approved by the Institutional Review Board of An‐Najah National University Hospital (IRB No. Pharm.Aug.2025/11). The study used anonymized retrospective data, and the requirement for informed consent was waived by the Institutional Review Board.

## 3. Results

### 3.1. Patient Characteristics and Baseline Comorbidities

Five consecutive dialysis‐dependent patients with MM who underwent ASCT during the study period were included in this case series. The mean age was 56 years (range, 51–68 years); three were men and two were women. All patients had dialysis‐dependent ESRD and received maintenance hemodialysis through permanent tunneled central venous catheters. Detailed patient‐level characteristics are provided in Supporting file 2.

Hypertension was present in all five patients. Other comorbidities included diabetes mellitus in one patient, respiratory disease in one, heart disease in two, and hepatitis B virus infection in one. The mean left ventricular ejection fraction was 57% (range, 55%–60%), and the mean length of hospitalization was 23.8 days (range, 16–45 days) (Supporting Table S2-1).

The mean duration of dialysis dependence before ASCT was 14.4 months (range, 4–37 months). The mean follow‐up duration after ASCT was 55.4 months (range, 21–80 months).

### 3.2. Pretransplant Characteristics and Transplant‐Related Variables

All five patients received antimyeloma therapy before ASCT. Two received VRD, two received VCD, and one received bortezomib alone; none had received radiotherapy. CD34+ cells were collected from peripheral blood in all patients. The mean CD34+ cell collection dose was 4.3 × 106/kg (range, 2.6–6.8 × 106/kg), and the mean infused dose was 4.1 × 106/kg (range, 2.3–6.8 × 106/kg). The mean interval from collection to reinfusion was 44.6 days (range, 18–85 days), and the mean interval from diagnosis to reinfusion was 217.6 days (range, 101–292 days) (Supporting Table S2-2).

### 3.3. Laboratory Profile

Expected postconditioning cytopenias were observed. The mean WBC count increased from 5.0 × 10^9^/L at admission to 7.5 × 10^9^/L at discharge, whereas the mean hemoglobin concentration decreased from 9.8 to 8.4 g/dL. The mean platelet count decreased from 284.0 × 10^9^/L at admission to 23.6 × 10^9^/L at discharge. This value represented the actual platelet count at discharge, not the nadir. All patients were clinically stable, had no active bleeding, and showed evidence of platelet recovery; therefore, discharge was considered appropriate with close outpatient follow‐up. The mean absolute neutrophil count increased from 3.7 to 4.7 × 10^9^/L. Renal indices remained elevated: mean creatinine changed from 5.7 to 6.1 mg/dL, and mean blood urea nitrogen changed from 38.8 to 40.9 mg/dL. C‐reactive protein increased during admission, consistent with the observed inflammatory and infectious burden, whereas alanine aminotransferase and alkaline phosphatase values remained stable (Supporting Table S2-3).

### 3.4. Hematologic Recovery and Complications

All patients achieved engraftment. Mean neutrophil engraftment occurred on Day 13.0 (range, 11–17 days), and mean platelet engraftment occurred on Day 19.4 (range, 15–26 days). The mean ANC nadir occurred on Day 8.0 (range, 6–12 days). The mean platelet nadir was 6.5 × 109/L (range, 2.0–21.6 × 109/L) and occurred on day 10.6 (range, 10–12 days). No graft failure occurred (Supporting Table S2-4).

Infectious complications were common. Four patients developed neutropenic fever. *Clostridioides difficile* infection was documented in three patients, and *Staphylococcus epidermidis* infection in two. Single cases of carbapenem‐resistant Enterobacterales, *Entamoeba histolytica*, and *Escherichia coli* infection were recorded. Documented infection sources included blood, urine, blood and urine, and combined blood, stool, and urine. Affected patients received broad‐spectrum antibacterial treatment, and antifungal agents were used according to the institutional protocol and clinical findings. Piperacillin–tazobactam was the most frequently administered antibacterial agent, followed by vancomycin and amikacin according to neutropenic pathway protocol; fluconazole and itraconazole were the most frequently administered antifungal agents (Supporting Table S2-5).

One patient (20%) experienced gastrointestinal bleeding. No thrombosis, acute liver injury, phlebitis, mechanical ventilation, or ICU admission was recorded. All patients survived beyond day + 100 after ASCT, and no 100‐day treatment‐related mortality occurred.

## 4. Discussion

This case series provides clinically relevant evidence that ASCT can be performed in carefully selected patients with MM and dialysis‐dependent ESRD. All five patients achieved neutrophil and platelet engraftment, and no treatment‐related mortality occurred within 100 days. These findings support the view that dialysis dependence should not automatically exclude an, otherwise, eligible patient from high‐dose melphalan conditioning followed by ASCT.

Our findings are consistent with retrospective studies of ASCT in patients with MM and severe renal impairment. A Russian single‐center study included 64 patients with MM and renal impairment; 23 were dialysis‐dependent at diagnosis, and 10 underwent ASCT while receiving hemodialysis [17], and a UK two‐center cohort evaluated 370 patients across a broad range of renal function [7]. Both studies supported the feasibility of ASCT in selected patients with renal impairment. In our cohort, melphalan was reduced to 140 mg/m^2^ for all patients, reflecting a cautious approach intended to limit toxicity in dialysis‐dependent ESRD.

The engraftment kinetics in our cohort were broadly comparable to those reported in previous studies. Published cohorts have generally shown timely neutrophil and platelet recovery after ASCT in patients with renal impairment [6]. Mean neutrophil and platelet engraftment occurred on Days 13.0 and 19.4, respectively. These observations suggest that dialysis dependence did not prevent hematopoietic recovery in this small cohort. The mean platelet count at discharge remained low; however, it represented the actual discharge value rather than the nadir. All patients were clinically stable, had no active bleeding, and showed an upward platelet‐recovery trend. Discharge decisions were, therefore, based on the overall clinical condition and recovery trajectory rather than a single predefined platelet threshold.

A practical contribution of this study is the detailed description of peritransplant care, particularly hydration and dialysis coordination. Intravenous 0.9% saline was administered for 2 h before and after melphalan on Day −2. Hemodialysis was performed approximately 24 h after melphalan administration, followed by autologous stem‐cell infusion 24 h later (approximately 48 h after melphalan), consistent with a previously reported protocol [18]. However, published dialysis schedules vary across transplant centers according to institutional protocols, pharmacokinetic considerations, and patient‐specific clinical factors, with some centers reporting substantially shorter intervals between melphalan administration and hemodialysis [19]. Such operational details are rarely reported but may be useful for transplant centers caring for similar patients, particularly where resources and access to specialized services are limited [16].

Infectious complications were the principal clinical challenge. Four patients developed neutropenic fever, two had CLABSIs, and two experienced septic shock. Previous studies have also identified substantial infectious risk after ASCT and among patients with long‐term hemodialysis access [16, 20]. The presence of a tunneled dialysis catheter, together with the need for intensive peritransplant vascular access, may increase the opportunity for catheter‐related infection. These findings emphasize meticulous catheter care, daily reassessment of line necessity, prompt culture collection, and early targeted antimicrobial therapy.

No patient became dialysis independent during the index hospitalization despite successful hematologic engraftment. Previous reports have described variable rates of dialysis independence after myeloma treatment and ASCT [7, 21]. In our cohort, creatinine and blood urea nitrogen remained elevated at discharge, and all patients continued maintenance hemodialysis. Differences between studies may reflect dialysis duration, reversibility of the underlying renal lesion, timing of effective myeloma therapy, and completeness of longitudinal renal follow‐up. Long‐standing ESRD and irreversible structural kidney damage may reduce the likelihood of renal recovery [2]. Recovery is more plausible when renal failure is recent and driven by potentially reversible processes such as cast nephropathy [22]. Earlier effective control of myeloma may, therefore, improve the opportunity for renal recovery in selected patients. The present findings also show that hematopoietic engraftment does not necessarily translate into immediate renal recovery [17, 19]. Complete longitudinal assessment is required to determine whether sustained disease control later reduces dialysis requirements.

The absence of 100‐day treatment‐related mortality is reassuring and is consistent with reports showing acceptable early mortality after ASCT in selected patients with renal impairment [16]. Similar findings have been reported in dialysis‐dependent and broader chronic kidney disease cohorts [7, 17]. Taken together, these data suggest that ASCT is not absolutely contraindicated by dialysis dependence when patients are carefully selected and supported by coordinated hematology, nephrology, infectious‐disease, and transplant‐nursing care.

The present study also adds evidence from a resource‐limited setting. It illustrates practical challenges involving infection prevention, dialysis scheduling, catheter management, and transfusion support. Although these findings are descriptive, they provide real‐world information that may assist transplant and nephrology teams working in similar settings [23].

This study has several limitations. The small sample size and single‐center design limit generalizability, and retrospective data collection may have resulted in incomplete or inconsistently documented variables. Although the duration of clinical follow‐up was available, longitudinal data on dialysis requirements, overall survival, progression‐free survival, and formal hematologic response were incomplete. Serum free light chain measurements were not consistently available; therefore, formal International Myeloma Working Group response categories could not be independently assigned, and the relationship between depth of myeloma response and renal outcome could not be evaluated. Renal status was assessed primarily during the index hospitalization, so delayed renal recovery could not be reliably determined. The study also lacked a comparator group and inferential analysis. Larger prospective multicenter studies using standardized hematologic and renal outcome definitions are needed.

In summary, dialysis dependence should not be considered an absolute contraindication to ASCT in patients with MM. Carefully selected patients can achieve timely engraftment and acceptable early outcomes; however, infectious complications, particularly those associated with vascular access, remain an important concern. Multidisciplinary care, meticulous infection prevention, coordinated dialysis, and longitudinal follow‐up are essential in this high‐risk population.

## 5. Conclusions

ASCT was feasible in five dialysis‐dependent patients with MM treated at a tertiary hospital in a low‐ and middle‐income country. All patients achieved hematologic engraftment, and no 100‐day treatment‐related mortality occurred. Neutropenic fever, CLABSI, and *Clostridioides difficile* infection were frequent complications. All patients remained dialysis dependent at discharge. Larger prospective studies with standardized long‐term hematologic and renal assessments are needed to identify patients most likely to achieve renal recovery.

## Author Contributions

M.B. and S.H.Z. conceived and designed the study. M.B., A.K., M.J.A., M.S., A.A.M.D., M.H., H.A., R.A., and A.E. contributed to data collection, curation, and validation. M.B., A.K., H.A., and R.A. contributed to the interpretation of the hematological and transplantation findings. M.H. and A.E. contributed to the interpretation of the renal and dialysis‐related findings. M.J.A., M.S., and A.A.M.D. conducted the literature review and assisted in preparing the manuscript. M.B. and S.H.Z. analyzed and interpreted the data. M.B. prepared the initial draft of the manuscript. S.H.Z. supervised the study and critically revised the manuscript. M.B. serves as the guarantor of the study.

## Funding

No specific funding was received for this study.

## Disclosure

All authors have reviewed and approved the final manuscript and agreed to be accountable for all aspects of the work.

## Ethics Statement

The study was approved by the Institutional Review Board of An‐Najah National University Hospital (IRB No. Pharm.Aug.2025/11). The requirement for informed consent was waived because the study used anonymized retrospective data.

## Consent

The manuscript contains no directly identifiable patient information. The requirement for consent was waived by the Institutional Review Board as part of the retrospective study approval.

## Conflicts of Interest

The authors declare no conflicts of interest.

## Patient Perspective

A patient perspective was not collected because this was a retrospective case series based on anonymized medical records.

## Supporting Information

Additional supporting information can be found online in the Supporting Information section.

## Supporting information


**Supporting Information 1** Supporting File S1: Joanna Briggs Institute (JBI) Critical Appraisal Checklist for Case Series.

**Supporting Information 2** Supporting File S2: Additional clinical and transplant‐related data for the included dialysis‐dependent patients. Supporting File S2 contains five tables: Table S2‐1, individual patient clinical summaries; Table S2‐2, baseline patient characteristics and transplant‐related variables; Table S2‐3, laboratory values at admission and discharge; Table S2‐4, hematologic recovery parameters; and Table S2‐5, post‐ASCT infectious complications and antimicrobial use.

## Data Availability

The datasets generated and analyzed during the current study are available from the corresponding author upon reasonable request.
