# Supplementary material for: Renal Outcomes Following Autologous Stem Cell Transplantation in Dialysis‐Dependent Patients With Multiple Myeloma: A Single‐Center Study From a Low‐ and Middle‐Income Country
Source: Case Rep Hematol. 2026 Sep 14;2026:1808249. doi: 10.1155/crh/1808249 (PMC13575482; doi:10.1155/crh/1808249)
Supplement: Supplementary file 1 [file CRH-2026-1808249-s001.docx]

Additional clinical and transplant-related data

**Table S2-1. Individual patient clinical summaries (n = 5)**

| **Patient** | **Age/sex** | **Diagnosis and timing** | **Comorbidities** | **Conditioning and vascular access** | **Post-ASCT course and discharge** |
| --- | --- | --- | --- | --- | --- |
| P1 | 51/male | Plasma-cell leukemia secondary to MM; diagnosed approximately 6–7 months before ASCT | ESRD on maintenance hemodialysis; hypertension | Melphalan 140 mg/m²; peripheral-blood CD34+ cells; tunneled dialysis catheter | Neutropenic fever and CLABSI caused by Staphylococcus epidermidis; discharged on day 16 |
| P2 | 52/female | MM; diagnosed approximately 9 months before ASCT | ESRD on maintenance hemodialysis; hypertension | Melphalan 140 mg/m²; peripheral-blood CD34+ cells; tunneled dialysis catheter | Neutropenic fever with typhlitis, C. difficile infection, amoebiasis, rectal CRE, and suspected gastrointestinal bleeding; discharged on day 17 |
| P3 | 57/female | MM; exact diagnosis date not available in the record | ESRD on maintenance hemodialysis; hypertension | Melphalan 140 mg/m²; peripheral-blood CD34+ cells; tunneled dialysis catheter | Neutropenic fever with negative cultures; received antibiotics, filgrastim, and blood-product support; discharged on day 18 |
| P4 | 68/male | MM; exact diagnosis date not available in the record | ESRD on maintenance hemodialysis; diabetes mellitus; hypertension | Melphalan 140 mg/m²; peripheral-blood CD34+ cells; tunneled dialysis catheter | Neutropenic septic shock due to CLABSI with E. coli and S. epidermidis; C. difficile infection; discharged on day 23 |
| P5 | 52/male | MM; diagnosed approximately 1 year before ASCT | ESRD on maintenance hemodialysis; hypertension; valvular heart disease | Melphalan 140 mg/m²; peripheral-blood CD34+ cells; right internal jugular tunneled dialysis catheter | Neutropenic fever with C. difficile infection; blood and urine cultures were negative; discharged on day 45 |

*Note: Exact diagnosis duration, individual pre-ASCT treatment regimen, and the etiology of ESRD were not consistently retrievable for every patient; only verified patient-level information is shown. Aggregate prior-treatment data are reported in Table 2.*

**Table S2-2. Baseline patient characteristics and transplant-related variables (n = 5)**

| **Variable** | **Category/value** | **n (%) or mean (range)** |
| --- | --- | --- |
| Sex | Male | 3 (60) |
|  | Female | 2 (40) |
| Diagnosis | Multiple myeloma or secondary plasma-cell leukemia | 5 (100) |
| Dialysis-dependent ESRD | Yes | 5 (100) |
| Hypertension | Yes | 5 (100) |
| Length of hospitalization, days |  | 23.8 (16–45) |
| Diabetes mellitus | Yes | 1 (20) |
| Respiratory disease | Yes | 1 (20) |
| Heart disease | Yes | 2 (40) |
| Hepatitis B virus infection | Yes | 1 (20) |
| Left ventricular ejection fraction, % |  | 57.0 (55–60) |
| Previous antimyeloma regimen | VRD | 2 (40) |
|  | VCD | 2 (40) |
|  | Bortezomib alone | 1 (20) |
| Prior radiotherapy | No | 5 (100) |
| Permanent tunneled dialysis catheter | Yes | 5 (100) |
| Conditioning regimen | Melphalan 140 mg/m² | 5 (100) |
| Reduced conditioning dose | Yes | 5 (100) |
| CD34 source | Peripheral blood | 5 (100) |
| Backup product | No | 5 (100) |
| CD34+ cell collection dose |  | 4.3 (2.6–6.8) × 10⁶/kg |
| CD34+ cell infusion dose |  | 4.1 (2.3–6.8) × 10⁶/kg |
| **Duration of dialysis dependence before ASCT, months** |  | **14.4 (4–37)** |
| **Follow-up duration after ASCT, months** |  | **55.4 (21–80)** |
| Time from collection to reinfusion, days |  | 44.6 (18–85) |
| Time from diagnosis to reinfusion, days |  | 217.6 (101–292) |

**Table S2-3. Laboratory values at admission and discharge (n = 5)**

| **Variable** | **Mean (range)** | |
| --- | --- | --- |
|  | **At admission** | **At discharge** |
| White blood cell count (× 10⁹/L) | 5.0 (4.1–7.0) | 7.5 (5.4–10.2) |
| Platelet count (× 10⁹/L) | 284.0 (200.0–373.4) | 23.6 (18.0–30.0) |
| Hemoglobin (g/dL) | 9.8 (8.7–11.2) | 8.4 (7.5–11.2) |
| Absolute neutrophil count (× 10⁹/L) | 3.7 (2.4–5.4) | 4.7 (1.0–8.0) |
| Blood urea nitrogen (mg/dL) | 38.8 (26.1–52.0) | 40.9 (29.0–47.8) |
| Creatinine (mg/dL) | 5.7 (3.6–7.9) | 6.1 (3.7–10.6) |
| C-reactive protein (mg/L) | 8.8 (0.5–26.0) | 40.2 (13.8–63.5) |
| Alanine aminotransferase (IU/L) | 13.9 (8.3–25.0) | 12.6 (6.0–25.0) |
| Alkaline phosphatase (IU/L) | 18.0 (12.8–25.0) | 14.8 (7.2–25.0) |

**Table S2-4. Hematologic recovery parameters (n = 5)**

| **Variable** | **Mean (range)** |
| --- | --- |
| Neutrophil engraftment (ANC ≥0.5 × 10⁹/L), day | 13.0 (11–17) |
| Platelet engraftment (platelet count ≥20 × 10⁹/L without transfusion), day | 19.4 (15–26) |
| ANC nadir, day | 8.0 (6–12) |
| Platelet nadir, day | 10.6 (10–12) |
| Platelet nadir (× 10⁹/L) | 6.5 (2.0–21.6) |

**Table S2-5. Post-ASCT infectious complications and antimicrobial use (n = 5)**

| **Variable** | **n (%)** |
| --- | --- |
| Neutropenic fever | 4 (80) |
| Septic shock | 2 (40) |
| CLABSI | 2 (40) |
| **Microbiologically documented infection** |  |
| Clostridioides difficile | 3 (60) |
| Staphylococcus epidermidis | 2 (40) |
| Carbapenem-resistant Enterobacterales | 1 (20) |
| Escherichia coli | 1 (20) |
| Entamoeba histolytica | 1 (20) |
| **Documented infection source** |  |
| Blood | 1 (20) |
| Urine | 1 (20) |
| Blood and urine | 1 (20) |
| Blood, stool and urine | 1 (20) |
| **Antimicrobial agents administered** |  |
| Piperacillin–tazobactam | 4 (80) |
| Vancomycin | 3 (60) |
| Amikacin | 2 (40) |
| Ceftriaxone | 1 (20) |
| Colistin | 1 (20) |
| Fluconazole | 3 (60) |
| Itraconazole | 2 (40) |
